# Transforming cataract care through artificial intelligence: an evaluation of large language models’ performance in addressing cataract-related queries

**DOI:** 10.3389/frai.2025.1639221

**Published:** 2025-09-05

**Authors:** Xinyue Wang, Yan Liu, Linghao Song, Yinuo Wen, Shenjie Peng, Ruoxi Ren, Yi Zhang, Tianhui Chen, Yongxiang Jiang

**Affiliations:** ^1^Eye Institute and Department of Ophthalmology, Eye and ENT Hospital, Fudan University, Shanghai, China; ^2^Key Laboratory of Myopia and Related Eye Diseases, NHC, Shanghai, China; ^3^Key Laboratory of Myopia and Related Eye Diseases, Chinese Academy of Medical Sciences, Shanghai, China; ^4^Shanghai Key Laboratory of Visual Impairment and Restoration, Shanghai, China; ^5^The First Affiliated Hospital of Zhejiang Chinese Medical University (Zhejiang Provincial Hospital of Chinese Medicine), Hangzhou, China

**Keywords:** large language models, cataract, patient education, artificial intelligence, cataract surgery

## Abstract

**Purpose:**

To evaluate the performance of five popular large language models (LLMs) in addressing cataract-related queries.

**Methods:**

This comparative evaluation study was conducted at the Eye and ENT Hospital of Fudan University. We performed both qualitative and quantitative assessments of responses from five LLMs: ChatGPT-4, ChatGPT-4o, Gemini, Copilot, and the open-source Llama 3.5. Model outputs were benchmarked against human-generated responses using seven key metrics: accuracy, completeness, conciseness, harmlessness, readability, stability, and self-correction capability. Additional inter-model comparisons were performed across question subgroups categorized by clinical topic type.

**Results:**

In the information quality assessment, ChatGPT-4o demonstrated the best performance across most metrics, including accuracy score (6.70 ± 0.63), completeness score (4.63 ± 0.63), and harmlessness score (3.97 ± 0.17). Gemini achieved the highest conciseness score (4.00 ± 0.14). Further subgroup analysis showed that all LLMs performed comparably to or better than humans, regardless of the type of question posed. The readability assessment revealed that ChatGPT-4o had the lowest readability score (26.02 ± 10.78), indicating the highest level of reading difficulty. While Copilot recorded a higher readability score (40.26 ± 14.58) than the other LLMs, it still remained lower than that of humans (51.54 ± 13.71). Copilot also exhibited the best stability in reproducibility and stability assessment. All LLMs demonstrated strong self-correction capability when prompted.

**Conclusion:**

Our study suggested that LLMs exhibited considerable potential in providing accurate and comprehensive responses to common cataract-related clinical issues. Notably, ChatGPT-4o achieved the best scores in accuracy, completeness, and harmlessness. Despite these promising results, clinicians and patients should be aware of the limitations of artificial intelligence (AI) to ensure critical evaluation in clinical practice.

## 1 Introduction

Nowadays, the increasing reliance of patients on social media and search engines for medical advice has rendered online health information seeking behavior (HISB) a ubiquitous global phenomenon (Zhang et al., 2021). Large language models (LLMs) chatbots, sophisticated artificial intelligence (AI) systems that possess the capacity for human-like text comprehension and generation, have become an increasingly popular modality for individuals seeking online health information (OHI). In the realm of ophthalmology, owing to the conversational interactivity and near-human-level performance in cognitive tasks, LLM-chatbots have the potential to address patient-specific questions (Antaki et al., 2024; Pushpanathan et al., 2023; Bernstein et al., 2023), and facilitate discussions on the diagnosis and treatments of ocular diseases (Thirunavukarasu et al., 2023; Alberts et al., 2023; Hu et al., 2023).

Unlike traditional supervised deep learning models, LLMs leverage self-supervised learning to efficiently acquire knowledge from vast amounts of unannotated data, and are fine-tuned on smaller annotated datasets to optimize performance on specific tasks defined by end-users5. Consequently, while chatbots can provide authoritative-sounding responses to complex medical queries, the reliability of their training data and processes is still a critical concern due to the risk of factually inaccurate responses (Chen et al., 2023; van Dis et al., 2023). The phenomenon of ‘hallucinations’ or ‘fact fabrication’, where inaccurate information is generated and presented, has been extensively documented (Chen et al., 2023; Ji et al., 2023; Alkaissi and McFarlane, 2023). For this reason, verifying the validity of the information provided by LLM-chatbots, particularly in the context of specialized ophthalmologic questions, is crucial to guarantee patient safety (Gupta et al., 2023).

A comprehensive patient counseling may be beneficial to help patients better prepare themselves for the surgery and reduce the anxieties that patients may experience preoperatively (Gupta et al., 2024; Ramirez et al., 2017; Newman-Casey et al., 2015). Despite the increasing prevalence of LLMs and their potential to assist patient education, the accuracy and utility of LLMs in the context of cataract care remain relatively unexplored. Furthermore, in addition to well-established closed-source LLMs such as ChatGPT and Copilot, Meta Platforms’ Llama-3.1405B (released in July 2024) has garnered significant attention for its enhanced language understanding, generation capabilities, and overall performance. As the first openly available model to rival leading AI models, its ability to provide accurate, comprehensive, and harmless information regarding cataract care-related queries remains uncertain, highlighting a critical gap in current research.

This study conducts a comprehensive evaluation of the performance of chatbot-generated responses to cataract-related queries, which are subjective, open-ended, and reflective of the challenges and ambiguities encountered by patients in clinical settings. By comparing the models’ response quality on cataract-related questions with OHI from authoritative ophthalmologic websites, this study provides an early evidence base on the reliability of chatbots in clinical settings. Furthermore, it highlights the limitations of LLM-generated medical information.

## 2 Methods

### 2.1 Question-answer database

This process began with systematic sourcing queries from authoritative OHI outlets, including the National Eye Institute, the American Academy of Ophthalmology, and the Eye and ENT Hospital of Fudan University. We focused on the most common and representative issues encountered by patients in clinical settings. The selected queries were then standardized through a careful process, ensuring that each question was framed clearly and consistently to reflect the most relevant and frequently addressed concerns in ophthalmology. Finally, a set of 104 questions was selected, covering potential concerns related to the pathophysiology, surgical procedure, postoperative care, and prognosis (Supplementary Table 1). From October 27th to December 25th, 2024, responses to these queries were generated by ChatGPT (version GPT-4 and GPT-4o, OpenAI), Gemini Advanced (Google LLC), Copilot (Microsoft Corp), and Llama-3.1405B (Meta Platforms). To promote clarity and coherence, the LLM-chatbots were instructed to respond in a consistently structured bullet-point format (Supplementary Table 2). Furthermore, each question was input as a standalone query to minimize potential memory retention bias and ensure that it was generated independently. The human comparator responses were developed through a dedicated clinical authorship initiative involving 20 experienced ophthalmologists from the Eye & ENT Hospital of Fudan University. These physicians created original responses based on firsthand clinical expertise and contemporary practice guidelines. Each response underwent standardization to ensure consistent structure and clinical applicability, with all outputs edited to maintain standard medical terminology. For evaluation, responses were subjected to blinded assessment, with all source identifiers removed.

### 2.2 Information quality assessment

The quality of all the responses was assessed for accuracy, completeness, conciseness, and harmlessness by a group of ophthalmologists, evaluated using a Likert scale, which aligns with a validated approach (Huang et al., 2024; Goodman et al., 2023). Supplementary Table 2 presents representative examples of LLM responses along with their corresponding evaluation scores. In order to further understand the strengths and weaknesses of the LLM-Chatbots in various subject matters, questions retrieved from websites were categorized into 9 domains—etiology (*N* = 12), symptoms (*N* = 8), diagnosis (*N* = 9), cataract surgery (*N* = 17), IOL-related (*N* = 12), postoperative care (*N* = 15), treatment and prevention (*N* = 11), PCO (*N* = 10), and prognosis (*N* = 10), and subgroup analysis was further conducted.

### 2.3 Readability assessment

A readability analysis was performed using Flesch Reading Ease and Flesch–Kincaid Grade Level. The readability scores ranged from 0 to 100, with higher scores demonstrating easier readability (Flesch, 1948). In contrast, a higher grade level corresponds to greater reading difficulty. Three additional metrics, including word count, sentence count, and syllable count, were compared for each group to show the response length of each LLM.

### 2.4 Reproducibility and stability assessment

To comprehensively evaluate model reproducibility and stability, all “cataract surgery” and “IOL-related” questions, regardless of initial scores, were regenerated and rescored using the five LLMs 30 days after initial answers were generated and scored. For responses generated by the LLM-Chatbots that received a poor accuracy (<5 on the accuracy scale), the LLM-Chatbots were further prompted to self-correct using this line “That does not seem quite right. Could you review?” (Lim et al., 2023). These revised responses were subsequently re-assessed for accuracy.

### 2.5 Likert scale definitions

Answer accuracy was measured on a 7-point Likert scale. Score 1 represented unacceptable inaccuracies; 2 to 3, poor accuracy with potentially harmful mistakes; 4, moderate inaccuracies that could be misinterpreted; 5 to 6, good quality with only minor, non-harmful inaccuracies; 7, very good accuracy that was devoid of any inaccuracies. A 5-point Likert scale (1: “not comprehensive/concise,” 2: “slightly comprehensive/concise,” 3: “moderately comprehensive/concise,” 4: “comprehensive/concise,” and 5: “very comprehensive/concise”) was used to evaluate the completeness and conciseness. A fourth metric, harmlessness, was also evaluated using a 5-point Likert scale (0: “not at all,” 1: “slightly,” 2: “moderately,” 3: “very,” and 4: “extremely”). The grading panel for this study comprised three experienced ophthalmologists. Methodological rigor was maintained through multiple raters and established evaluation criteria to minimize potential bias. We also used randomization in the response order to reduce bias.

### 2.6 Statistical analysis

Due to the ordinal nature of Likert scale data and the non-normal distribution of the data, score results were presented descriptively with median [IQR] values. Nonparametric tests, specifically the Mann–Whitney U test and the Kruskal-Wallis test, were used to determine differences in quality metrics, including accuracy, conciseness, and harmlessness, as well as readability metrics between different groups, followed by Bonferroni *post-hoc* test. Response agreement was graded using the Wilcoxon matched-pairs signed rank test and weighted *κ* statistic across all scores (1–7 for accuracy) to evaluate reproducibility and stability. A two-sided *p* < 0.05 was considered statistically significant. GraphPad Prism 9.5 (GraphPad Software, California, USA) and SPSS software version 26.0 (IBM Corp, Armonk, NY) were used for all analyses.

## 3 Results

### 3.1 Information quality assessment

**Figure 1:**
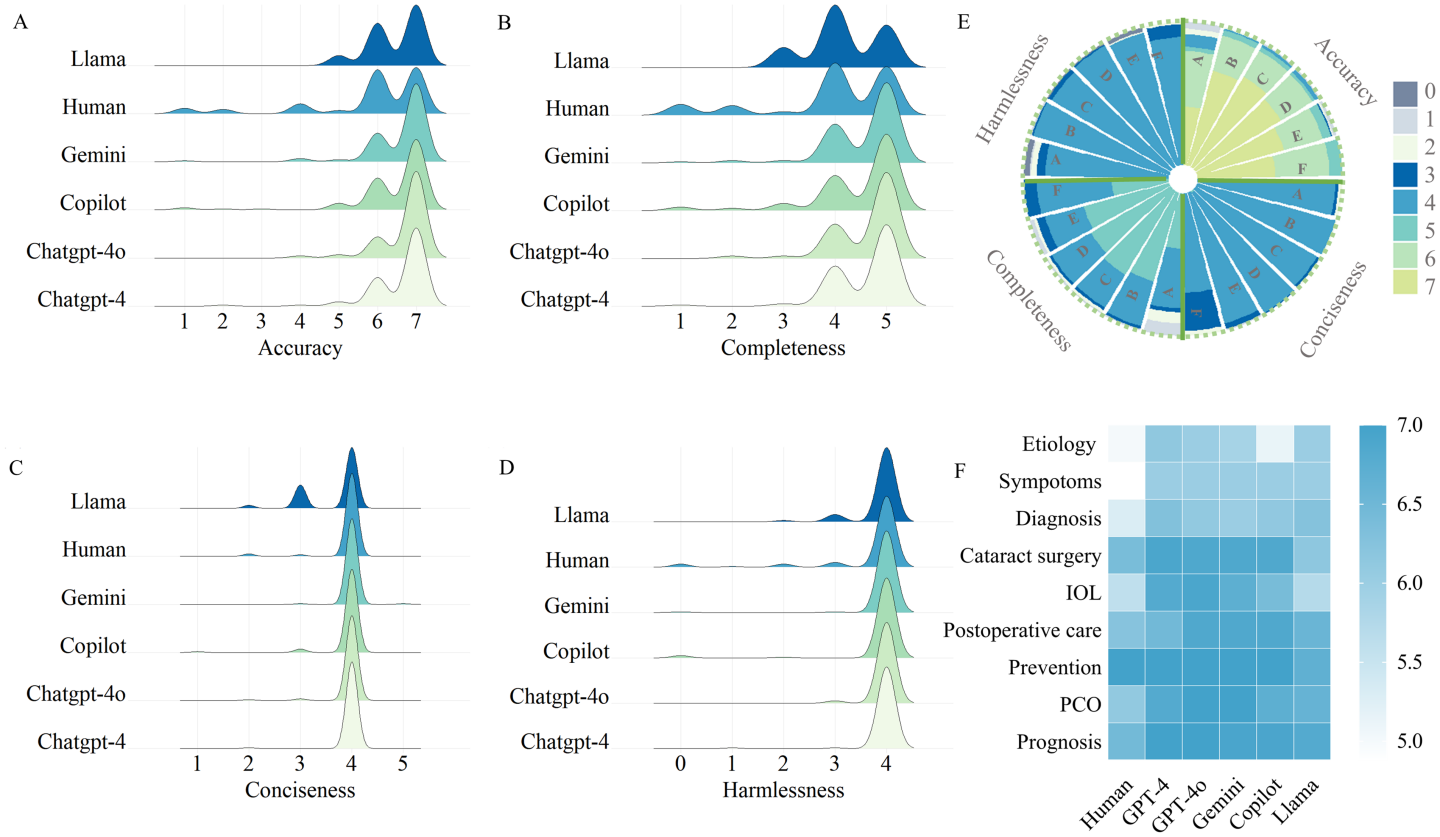
Evaluation of Chatbot-generated and human responses. **(A)** Consensus-based accuracy score of LLM-Chatbot responses to cataract care-related questions. **(B)** Consensus-based completeness score of LLM-Chatbot responses to cataract care-related questions. **(C)** Consensus-based conciseness score of LLM-Chatbot responses to cataract care-related questions. **(D)** Consensus-based harmlessness score of LLM-Chatbot responses to cataract care-related questions. **(E)** Grouped Stacked Columns of the scores of LLM-Chatbot responses. **(E)** LLMs’ performance in special domain of cataract care.

Figure 1A illustrates the consensus-based accuracy scores of LLM-Chatbots’ responses to cataract-related questions assessed by ophthalmologists. Human demonstrated an average accuracy score of 5.81 ± 1.62, inferior to all the closed-source LLMs, including ChatGPT-4 (6.59 ± 0.76; Bonferroni *post-hoc* test, *p* < 0.001), ChatGPT-4o (6.70 ± 0.63; Bonferroni *post-hoc* test, *p* < 0.001), Gemini (6.56 ± 0.87; Bonferroni *post-hoc* test, *p* < 0.001), and Copilot (6.40 ± 1.12; Bonferroni *post-hoc* test, *p* = 0.008). Although compared to the closed-source LLMs, Llama 3.1 exhibited a lower average accuracy score of 6.45 ± 0.66, it demonstrated accuracy comparable to that of human in answering cataract-related questions (Bonferroni *post-hoc* test, *p* = 0.722).

For a more detailed exploration of the quality of the responses generated by LLMs, Figures1B–E and Supplementary Table 3 exhibited the scores for comprehensiveness, conciseness, and harmlessness. All the LLM-Chatbots demonstrated optimal performance, with mean scores exceeding 4 out of a maximum of 5, for both completeness and conciseness. Regarding harmlessness, LLM-Chatbots achieved perfect scores for the majority of questions, indicating the safety of using LLM-Chatbots for cataract-related queries. Performance was consistent across ChatGPT-4, ChatGPT-4o, Gemini, and Copilot, with no significant statistical differences observed. However, Llama performed less favorably than the closed-source LLMs in certain categories such as “cataract surgery” and “prognosis.”

Figure 1F and Supplementary Table 4 provide a detailed subgroup analysis of the accuracy scores across the nine cataract care domains. Overall, no significant difference was found between the four closed-source LLMs in any domain. Furthermore, all of the groups performed consistently well in the domains of ‘Postoperative care’ and ‘Treatment and prevention’, achieving a median score of 7. In the ‘Prognosis’ and ‘PCO’ domain, five LLMs performed optimally, receiving greater accuracy scores compared to human (Kruskal-Wallis, *p* < 0.001). However, in the ‘cataract surgery’, and ‘IOL-related’ domains, the open-resource LLM Llama performed less optimally than other groups (Kruskal-Wallis, *p* < 0.001).

### 3.2 Stability and self-correction capabilities

Among all the five LLM-Chatbots, Copilot shows the best stability, with a median accuracy score of 7.0 [IQR, 7.0–7.0] for the first answers, and also 7.0 [IQR, 7.0–7.0] for rescored answers (*p* = 0.317 determined by Wilcoxon matched-pairs signed rank test). There was great interrater agreement for accuracy (weighted *κ* = 0.807; *p* < 0.001) (Landis and Koch, 1977). In terms of completeness, conciseness and harmlessness, Copilot gained totally the same scores on the same questions. With poor interrater agreement for accuracy (*p* = 0.059 determined by Wilcoxon matched-pairs signed rank test; weighted *κ* = 0.258; *p* = 0.009), Gemini showed the worst stability. Table 1 and Supplementary Table 5 presents the detailed results of the consistency and pairwise tests, illustrating the stability of all the LLM-Chatbots. Table 2 demonstrates the LLM-Chatbots’ ability to self-correct when prompted. Overall, all LLM-Chatbots exhibited substantial self-correction capabilities.

**Table 1:** The stability of the LLMs.

LLM	First score	Second score	*p*^a^ value	κ	95% CI	*p*^b^ value
ChatGPT-4	7.0 [7.0–7.0]	7.0 [7.0–7.0]	0.126	0.552	(0.184, 0.920)	< 0.001
ChatGPT-4o	7.0 [7.0–7.0]	7.0 [7.0–7.0]	0.223	0.529	(0.062, 0.947)	< 0.001
Gemini	7.0 [6.0–7.0]	7.0 [6.0–7.0]	0.059	0.258	(0.094, 0. 458)	0.009
Copilot	7.0 [7.0–7.0]	7.0 [7.0–7.0]	0.317	0.807	(0.591, 0.996)	< 0.001
Llama	7.0 [6.0–7.0]	7.0 [6.0–7.0]	0.245	0.606	(0.368, 0.844)	< 0.001

a *p* value determined by Wilcoxon matched-pairs signed rank test.

b *p* value determined by weighted kappa.

*LLM, Large Language Model.

**Table 2:** Demonstration of LLMs’ ability to self-correct when prompted.

LLM	Question	Initial	Self-corrected
ChatGPT-4	Are there alternatives to eyedrops after cataract surgery for people having difficulty putting in their eyedrops?	1	6
ChatGPT-4o	Do IOLs never need to be replaced?	3	4
Gemini	As a child’s eyes continue to develop, will the IOL need to be replaced in the future?	3	7
ChatGPT-4o	Can children with congenital cataracts be managed conservatively until they are older before undergoing surgical intervention?	3	7
Copilot	Is it true that children’s poor eyesight is due to their eyeballs not being fully developed, and that it will gradually improve?	2	6
Copilot	What’s the best treatment for cataracts?	2	6
Copilot	Will my IOL correct my lazy eye after cataract surgery?	2	7
Copilot	If cataract surgery is performed without implantation of an artificial intraocular lens (IOL), does this indicate surgical failure?	1	7

*LLM, Large Language Model; IOL, Intraocular Lens.

### 3.3 Readability

**Figure 2:**
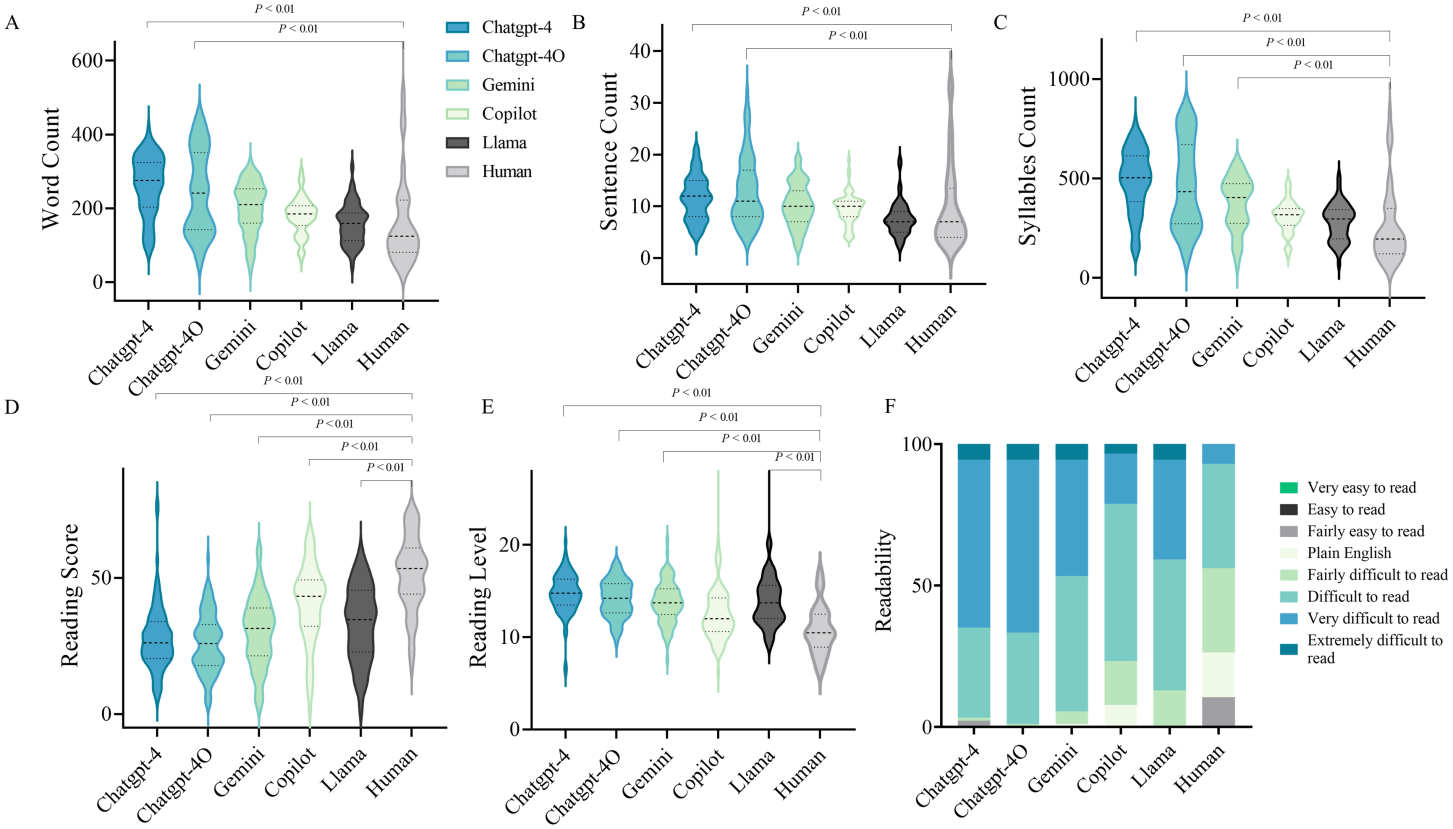
Readability evaluation of the LLMs. **(A)** Word count of LLM-Chatbot generated responses to cataract care-related questions. **(B)** Sentence count of LLM-Chatbot generated responses to cataract care-related questions. **(C)** Syllables Count of LLM-Chatbot generated responses to cataract care-related questions. **(D)** Reading score of LLM-Chatbot generated responses. **(E)** Reading level of LLM-Chatbot generated responses. **(E)** Grouped Stacked Columns of the readability of LLM-Chatbot responses.

Figures 2A–C and Supplementary Table 6 present the length of the LLM-Chatbots’ responses to the 104 selected cataract-related questions. Notably, both ChatGPT-4o and ChatGPT-4 exhibited significantly higher average totals in word, sentence, and syllable counts compared to human responses, indicating significantly longer response lengths. Furthermore, the mean readability score for human answers was 51.54 ± 13.71, which was significantly higher than that of LLMs, including ChatGPT-4 (27.83 ± 12.19, *p* < 0.001), ChatGPT-4o (26.02 ± 10.78, *p* < 0.001), Gemini (30.27 ± 12.73, *p* < 0.001), Copilot (40.26 ± 14.58, *p* < 0.001), and Llama (33.27 ± 13.69, *p* < 0.001), indicating a lower Flesch–Kincaid Grade Level for human responses (Figures 2D,E). Figure 2F presents a stacked bar chart illustrating the proportions of responses across various readability levels. This visualization provides deeper insight into the nuanced performance of the LLMs in terms of readability.

## 4 Discussion

LLMs are transforming the manner in which patients access and engage with broadly available medical information (Clusmann et al., 2023; Tailor et al., 2024). Instead of interacting with healthcare professionals or conducting extensive online searches, users are increasingly turning to LLMs to pose questions and receive direct responses. Given the propensity of LLMs to generate answers that may lack reliable sources or contain inaccuracies and potentially false citations, coupled with their variable accuracy, it is imperative for ophthalmologists to develop a comprehensive understanding of these models. Consequently, it becomes critical to evaluate the relevance and precision of LLM-generated responses to ophthalmologic inquiries within real-world contexts.

Previous researches have highlighted that the utilization of LLMs can be advantageous in various aspects of patient management and information dissemination within the field of ophthalmology (Bernstein et al., 2023; Dihan et al., 2024). However, in the domain of cataract, the research results do not seem to be very optimistic. Moshirfar et al. (2023) have demonstrated that while GPT-4 outperformed both GPT-3.5 and human experts when addressing the ophthalmological questions from StatPearls in most categories, it was found to be less effective than human professionals specifically in the category of “lens and cataract” (Moshirfar et al., 2023). Additionally, another study has indicated that the accuracy of ChatGPT’s responses regarding cataract surgery is inconsistent, varying with the nature of the query. ChatGPT achieved an optimistic accuracy score when detailing the procedural steps, lens options, and refractive outcomes of cataract surgery. However, its accuracy decreased when describing the risks and benefits associated with the procedure (Gupta et al., 2024). Existing studies predominantly rely on relatively small sample sizes and offer limited comprehensiveness in evaluating the performance metrics of LLMs, with a notable deficiency in the depth and detail of related investigations.

**Figure 3:**
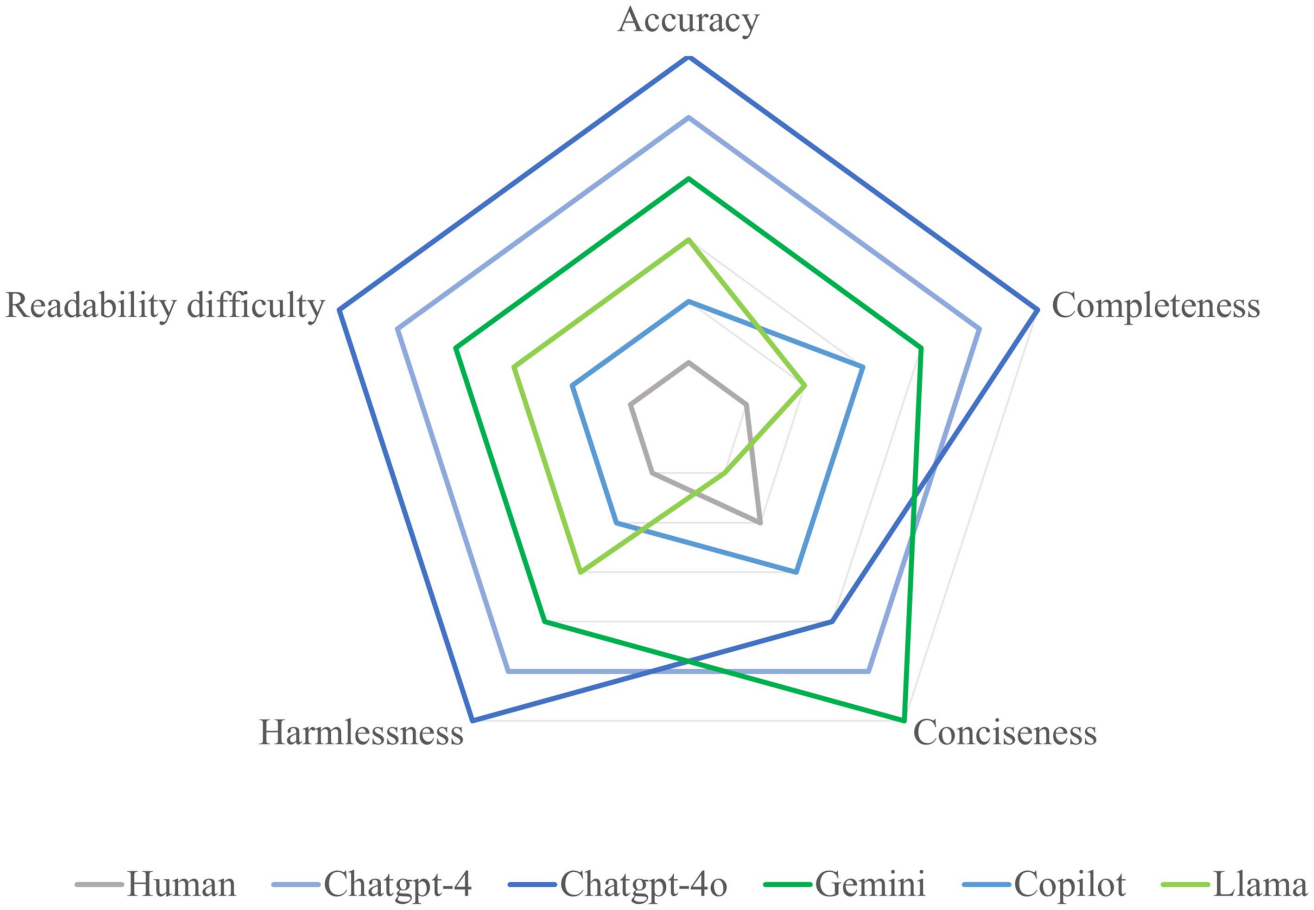
Radar chart demonstrated the overall performance of the LLMs.

This study conducted a qualitative and quantitative assessment of the appropriateness of responses from the five most popular LLMs concerning cataract-related clinical inquiries across multiple dimensions. The findings revealed that closed-source LLMs exhibited robust aggregate appropriateness, outperforming both human responses and open-source models across various domains. Among the evaluated LLMs, ChatGPT-4o distinguished itself as the most adept in addressing cataract-related questions, attaining the best performance across all assessment metrics (Figure 3). In contrast, since the LLMs were not specifically trained for this particular purpose (Sandmann et al., 2024), the open-source LLM Llama, despite showing comparable competence in delivering comprehensive responses, generally fell short of the performance observed in closed-source LLMs. This limitation highlights significant concerns regarding the efficacy of LLMs, particularly open-source models. Such concerns warrant careful scrutiny in the domain of cataract care, as the reliability and accuracy of these models are essential for their effective use in clinical practice. Regarding readability, AI-generated responses demonstrated significantly higher text complexity than human-generated content. This poses comprehension challenges—particularly for vulnerable populations like the elderly or those with limited health literacy. Such complexity carries clinical significance, as reduced readability could impede patients’ understanding of medical information, potentially influencing clinical decision-making—a consideration warranting attention in ophthalmic practice. Additionally, all LLM-chatbots exhibited substantial self-correction capabilities. In the stability assessment, the evaluated LLMs, except for Gemini, demonstrated moderate to strong stability in their performance, further indicating their reliability in providing responses to cataract-related inquiries.

The enhanced performance observed in this study, compared to previous evaluations, can be attributed to refined prompting techniques that specifically directed the model to respond in the format of an ophthalmology note while also instructing the LLM chatbots to present their responses in a structured bullet-point format, enhancing clarity and coherence. It is essential for clinicians and patients to recognize that the quality of LLM responses can be significantly influenced by user prompts. Well-defined prompts with specific instructions are considerably more effective in eliciting accurate and precise responses (Young and Zhao, 2024).

This investigation demonstrates multiple strengths. We rigorously evaluated five LLMs in their responses to common cataract-related queries. A robust methodological framework, incorporating randomization and meticulous appraisal by consultant ophthalmologists, ensured the integrity of the assessments. Notwithstanding these contributions, several limitations should be acknowledged. First, qualitative evaluations by experts entail inherent subjectivity. To address this, experienced ophthalmologists employed standardized criteria and consensus-based ratings to enhance objectivity. Second, because the analysis focused on the most prevalent patient-centered cataract concerns and relied on English for both query formulation and response generation, it necessarily excluded specialized topics such as rare complications. Moreover, the distribution of questions across domains was uneven (for instance, only 10 queries related to PCO), and these linguistic and sampling constraints may introduce bias and diminish statistical power. Consequently, domain-specific findings should be interpreted cautiously and validated using larger, more balanced datasets, alongside personalized clinical approaches to address complex knowledge gaps. Additionally, LLM performance is highly sensitive to prompt engineering, underscoring the necessity for rigorous standardization frameworks before clinical deployment. Given the rapid evolution of LLM technology, continuous evaluation aligned with technological developments is critical to maintain relevance. Taken together, these considerations highlight the need for ongoing validation as language models and clinical applications continue to evolve.

## 5 Conclusion

Taken together, our findings indicate that LLM-chatbots, particularly ChatGPT-4o, possess the potential to deliver accurate and comprehensive responses to cataract-related inquiries. In further assessments, LLMs exhibited commendable capabilities in various dimensions, including conciseness, safety, stability, and self-correction. However, regarding readability, it was observed that the complexity of their responses may present a higher level of difficulty compared to human-generated content, potentially necessitating a certain level of specialized knowledge for adequate comprehension. The implications of our findings are profound, as they suggest a viable pathway for the incorporation of LLM chatbots into cataract care management, potentially improving patient engagement and information accessibility. Furthermore, both patients and clinicians must remain cognizant of the inherent limitations of these LLMs, fostering an environment of informed usage and critical evaluation in clinical practice.

## Data Availability

The original contributions presented in the study are included in the article/Supplementary material, further inquiries can be directed to the corresponding authors.
