# Supplementary material for: Transforming cataract care through artificial intelligence: an evaluation of large language models’ performance in addressing cataract-related queries
Source: Front Artif Intell. 2025 Sep 5;8:1639221. doi: 10.3389/frai.2025.1639221 (PMC12447166; doi:10.3389/frai.2025.1639221)
Supplement: Supplementary file 1 [file Data_Sheet_1.DOCX]

Supplementary Material

# Supplementary Tables

**Supplementary Table 1.** Question Database.

**Supplementary Table 2.** Representative LLM response examples.

**Supplementary Table 3.** Ophthalmologist evaluation of Chatbot-generated and human-written Responses.

**Supplementary Table 4.** The overall quality of the responses generated by LLMs across nine domains.

**Supplementary Table 5.** Inter-rater agreement for completeness, conciseness, and harmlessness.

**Supplementary Table 6.** Overview of response length from LLM-Chatbots to cataract care-related questions.
